# In Silico Discovery of Candidate Drugs against Covid-19

**DOI:** 10.3390/v12040404

**Published:** 2020-04-06

**Authors:** Claudia Cava, Gloria Bertoli, Isabella Castiglioni

**Affiliations:** 1 Institute of Molecular Bioimaging and Physiology, National Research Council (IBFM-CNR), Via F.Cervi 93, 20090 Segrate-Milan, Milan, Italy; 2 Department of Physics “Giuseppe Occhialini”, University of Milan-Bicocca Piazza dell’Ateneo Nuovo, 1 - 20126, Milan, Italy; isabella.castiglioni@ibfm.cnr.it

**Keywords:** bioinformatics, covid-19, drugs, gene network

## Abstract

Previous studies reported that Angiotensin converting enzyme 2 (ACE2) is the main cell receptor of SARS-CoV and SARS-CoV-2. It plays a key role in the access of the virus into the cell to produce the final infection. In the present study we investigated in silico the basic mechanism of *ACE2* in the lung and provided evidences for new potentially effective drugs for Covid-19. Specifically, we used the gene expression profiles from public datasets including The Cancer Genome Atlas, Gene Expression Omnibus and Genotype-Tissue Expression, Gene Ontology and pathway enrichment analysis to investigate the main functions of *ACE2*-correlated genes. We constructed a protein-protein interaction network containing the genes co-expressed with *ACE2*. Finally, we focused on the genes in the network that are already associated with known drugs and evaluated their role for a potential treatment of Covid-19. Our results demonstrate that the genes correlated with *ACE2* are mainly enriched in the sterol biosynthetic process, Aryldialkylphosphatase activity, adenosylhomocysteinase activity, trialkylsulfonium hydrolase activity, acetate-CoA and CoA ligase activity. We identified a network of 193 genes, 222 interactions and 36 potential drugs that could have a crucial role. Among possible interesting drugs for Covid-19 treatment, we found Nimesulide, Fluticasone Propionate, Thiabendazole, Photofrin, Didanosine and Flutamide.

## 1. Introduction

Several members of the Coronaviridae family cause mild respiratory disease; two members of this family, the Severe Acute Respiratory Syndrome Coronavirus (SARS-CoV) and the Middle East Respiratory Syndrome Coronavirus (MERS-CoV), affect species from animals to humans, causing serious forms of respiratory disease. SARS-CoV appeared in 2002 in Guandong province (China) and its circulation caused more than 8000 cases, with 774 dead individuals [1]. Nowadays, no specific antiviral treatment exists to defeat this disease that was previously ended by control measures, such as travel restriction and patient isolation.

In winter 2019 a new form of pneumonia disease emerged in Wuhan, Hubei province (China) [2,3,4]. It was called SARS-CoV-2 (causing the coronavirus disease 2019, COVID-19) and rapidly spread from animals (pangolins or bats as possible sources) to humans.

The diffusion in humans was very rapid. On April 1st, 2020, a total of 75,4948 confirmed infections were reported worldwide, with 36,571 deaths [5].

The World Health Organization (WHO) strategy to contain the spreading includes the reduction of human-to-human spreading by limiting the contact between individuals, thus preventing transmission amplification events and communicating critical risk information to all communities [5].

While the diagnosis of COVID-19 is based on the amplification of the viral genome in real-time PCR with specific probes, the current treatment of affected individuals is limited to a mixture of a broad-spectrum of antiviral drugs [6]. However, in many cases this pharmacological approach has proven to be totally ineffective.

Screening drug studies on pangolin SARS-CoV-2, which is the human-related Coronavirus, demonstrated that three drugs (cepharanthine, selamectin and mefloquine hydrochloride) were effective in inhibiting viral replication, with cepharanthine potently inhibiting coronavirus infection at viral entry and post-entry viral replication [7]. The last drug is an anti-inflammatory and antineoplastic alkaloid approved for leukopenia and it is also proposed to inhibit the human immunodeficiency virus type 1 (HIV) entering in cells by reducing plasma membrane fluidity. In humans, it has a reduced toxicity.

Mefloquine is the approved treatment for malaria and has antiviral activity against both MERS-CoV and SARS-CoV [8]. The antiviral mechanism of selamectin is still unknown, as it is used usually as a topical broad-spectrum parasiticide in little animals (e.g., cats and dogs).

The entry of the virus in the cells is mediated by spike (S) glycoprotein; in particular, the spike 1 (S1) surface unit allows the attachment of the virus to cellular receptors. To allow the entry of the viral particles, the S protein is cleaved by cellular proteases at the S1/S2 and the S2′ site. Then, the viral capside is fused with the cellular membrane, a process driven by the S2 subunit [9]. It has been described that SARS-CoV entrance is mediated by angiotensin-converting enzyme 2 (ACE2) [10], and that the serine protease TMPRSS2 is responsible for the S protein cleavage [11,12]. The analysis of the sequences of the receptor binding motif (RBM) within the receptor binding domain revealed that it is responsible for the binding to ACE2 and that SARS-CoV and SARS-CoV-2 have conserved residues, suggesting that their binding with ACE2 could be similar, while the same residues are absent in other coronaviruses [13,14]. Moreover, some antibodies developed against human ACE2 blocked SARS-CoV and SARS-CoV-2 infections.

ACE2 belongs to the renin-angiotensin-aldosterone system (RAAS), which plays important roles in regulating blood pressure and body fluid, contributing to the pathophysiology of hypertension and cardiovascular/renal diseases by maintaining homeostasis of blood pressure, electrolyte balance and inflammatory responses. The protease Renin, generated mainly in the kidney, cleaves angiotensinogen to generate Angiotensin I (AngI); the angiotensin-converting enzyme 2 (ACE2) cleaves Ang I to produce Ang II, a key effector of the RAAS. AngII induces activation of Ang II type 1 and 2 receptors (AT1R and AT2R) in order to obtain vasoconstriction and inactivation of vasodilator bradykinin by cleavage. ACE2 is a terminal carboxypeptidase, a type I transmembrane glycoprotein, and a potent negative regulator of RAAS, localized on the apical surface of well-differentiated airway epithelia, especially ciliated cells [15].

Attenuation of ACE2 catalytic function alters RAAS system activity, resulting in enhanced inflammation and vascular permeability observed in the pathogenesis of inflammatory lung disease [16]. Indeed, when AT1R and AT2R are activated, they lead to the increased expression of proinflammatory mediators such as interleukin-8/Cytokine-induced Neutrophil Chemoattractant-3 and interleukin-6, triggering an inflammatory process in the lungs and other organs. In particular, AT1R activates NFkB transcription factor and AP-1, which in turn increases cytokine expression, apoptosis, vasoconstriction, fibroproliferation, the retention of Na+, and the enhancement of lung injury. The endogenous Ang II inhibits alveolar fluid clearance (AFC) and dysregulates ENaC expression via AT1R, contributing to alveolar filling and pulmonary edema.

ACE2 enzyme is expressed in a very small part of the lung population: the type II alveolar cells (AT2) that are about 0.64% of the lung cells. Single-cell RNA sequencing reveals that the ACE2 is also expressed in type I alveolar cells (AT1) cells, airway epithelial cells, fibroblasts, endothelial cells, and macrophages. Bioinformatics tools give quantitative information at single-cell resolution and reveal that Asian subjects have a much higher ACE2-expressing cell ratio than white and African American subjects, with males more affected than females. Abundant expression of ACE2 in a population of AT2 explained the severe alveolar damage after infection [17].

Experimental approaches for the study of interactions between drug compounds and target proteins are costly and time consuming. Computational approaches offer methods to test hypotheses of new putative drugs, reducing the cost and shortening the time. In particular, the identification of virus-associated protein-protein interactions offer evidences that could elucidate the mechanisms of viral infections. This methodology also allowed researchers to identify several candidate drugs for MERS-CoV, Ebola virus and Zika virus [18,19,20].

In this study, in order to investigate the molecular mechanism of ACE2 in COVID-19, we explored the *ACE2* expression in normal lung tissue based on the public RNA-seq profiles from The Cancer Genome Atlas (TCGA). In particular, we focused on the gene network correlated with *ACE2* expression in order to identify in silico all the interactors of *ACE2* that could attend to the viral infection in lung tissue. Then, we analyzed which drugs could interact with the genes of the network in order to identify new potentially effective drugs with antiviral properties.

## 2. Materials and Methods 

### 2.1. Public Datasets 

To obtain a clear view of the genes in the respiratory tract, RNA-seq data of normal lung tissues was extracted from the The Cancer Genome Atlas Lung Adenocarcinoma (TCGA-LUAD project. We downloaded, normalized, and filtered RNA-seq raw counts of 58 normal lung tissue samples using the reference of hg19, following the pipeline of the R/ Bioconductor package TCGAbiolinks [21].

Two Gene Expression Omnibus (GEO) datasets, GSE994 and GSE17913, were analyzed from the Gene Expression Omnibus (GEO) database (https://www.ncbi.nlm.nih.gov/geo/). GSE994 contains gene expression profiles from bronchial Epithelium tissues of 23 non-smoking volunteers; GSE17913 contains transcriptomic profiles from the oral mucosa of 40 non-smoking volunteers. From the Genotype-Tissue Expression (GTEx) project we considered the lung tissue-specific gene expression of 320 healthy volunteers.

### 2.2. Correlation, Gene Ontology and Enrichment Analysis

We performed a correlation analysis between *ACE2* and the other genes in TCGA-LUAD to obtain a network of all the possible *ACE2*-interactors. For each gene, we calculated the Pearson’s correlation with *ACE2* expression level. Considering the corresponding *p*-values of the correlation, only *ACE2* and genes significantly correlated (*p*-value < 0.01) were considered for the subsequent analysis [22].

We performed a Gene Ontology and a pathway analysis using the list of correlated genes to identify the functional role of the genes of the network [23]. Gene Ontology (GO) is a standardised annotation of gene products used to investigate the biology of a gene product in any organism. There are three GO categories that describe the function of a gene product at the molecular level, the biological process in which the gene product participates and the cellular component where the gene product is localized. In this way, we defined the categories (GO: biological process, GO: cellular components, GO: molecular function) to which each gene of the network belongs to [23].

In the second step, a pathway enrichment analysis using Fisher’s test was performed to assess whether there is an over-representation of correlated genes within given pathways [24,25]. If this happens, it means that this pathway is particularly important within the network of *ACE2* and its co-expressed interactors. We considered the pathways enriched with correlated genes if FDR < 0.01. *p*-values were adjusted with the Benjamini-Hochberg procedure for multiple testing correction [26].

### 2.3. Protein-Protein and Drug Interaction

We constructed a protein-protein interaction (PPI) network from the correlated genes. PPIs, which contain physical interactions, were downloaded using SpidermiR [27]. Specifically, we generated a network that involves the direct interactions among *ACE2*-correlated genes according to the PPI network. We verified if the genes within the network were already associated with known drugs and evaluated their role in the network [28]. The interaction between the drug and protein was obtained using the Matador and DGIdb database [29,30]. We integrated drug-gene interactions in the network using Fisher’s Test to verify if there is an over-representation of drug target genes in the network. We considered that the only significant interactions that obtained a FDR < 0.05. *p*-values were adjusted with Benjamini-Hochberg procedure for multiple testing correction [26]. In this way, we generated a map of drugs acting on the *ACE2* and *ACE2*-correlated network.

## 3. Results

**Figure 1:**
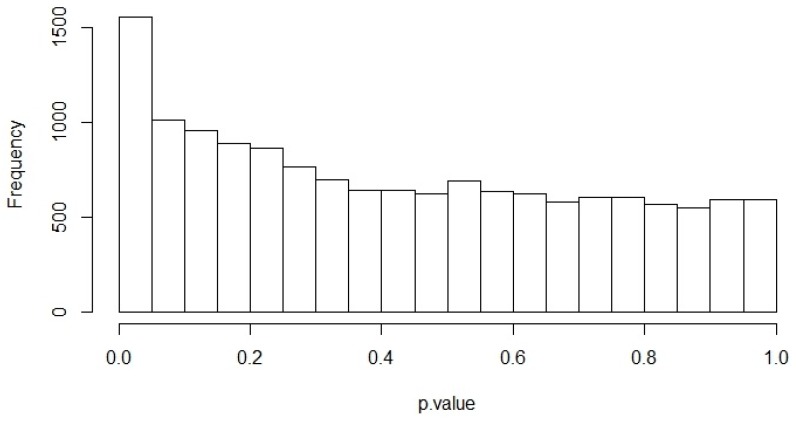
Correlation analysis between *ACE2* expression level and 14,700 genes in the 58 normal lung samples. Distribution of *p*-values from Pearson’s Correlation test.

The selection procedures in the pre-processing step allowed us to obtain 14,701 genes from the RNA-seq data analysis of lung normal tissues downloaded from the TCGA-LUAD project. The correlation analysis was applied between ACE2 expression level and 14,700 genes in the 58 lung normal samples. Figure 1 shows the *p*-value histogram originated from Pearson’s Correlation test for all gene pairs (ACE2 with the other 14,700 genes). *p*-value distributions were close to the uniform distribution between 0 and 1. The peak close to 0 indicates low *p*-values.

We obtained 526 genes that correlated with *ACE2* expression levels (*p*-value <0.01). This is the basic ACE2-correlated gene network on which we performed further analysis. The supplementary data 1 shows the 526 *ACE2* correlated genes.

Among the top 10 genes with a more significant p-value, we found that nine genes (*LRRK2, ACSL5, HSD17B4, EPHX1, MCCC2, GSTA4, ACACA, HGD* and *ROS1*) positively correlated with *ACE2* and one gene (*CRIP2*) negatively correlated (Table 1).

**Figure 2:**
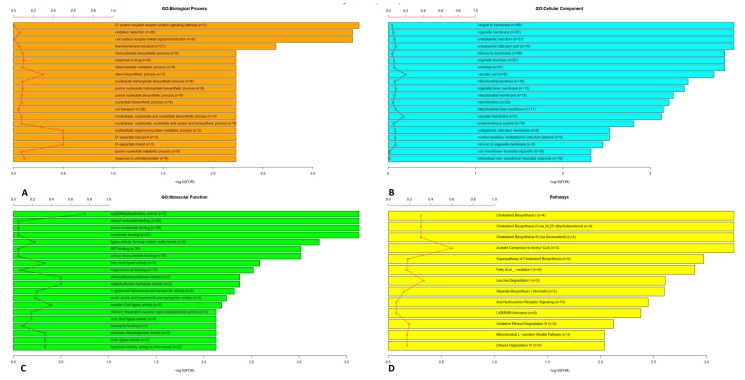
Top 20 Gene Ontology categories: Biological Process (BP) (**A**), Cellular Component (CC) (**B**), Molecular Function (MF) (**C**) and Pathways (**D**) enriched by correlated genes with *ACE2*, respectively. The red line represents the percentage between the number of genes related to *ACE2* in that category and the total number of genes in the category.

We evaluated the roles of the 526 genes using GO and pathway enrichment analysis. Figure 2 shows the bar chart with the numbers of genes assigned to the main categories of three ontologies, namely GO: biological process (Figure 2A), GO: cellular component (Figure 2B) and GO: molecular function (Figure 2C). We observed that the network contains genes that belong to the sterol biosynthetic process, multicellular organism protein metabolic process, D-aspartate transport and the import and response to unfolded protein. Moreover, the genes of the network encoded for protein of the vacuolar part and vacuolar membrane. Finally, among their activity, they were mainly involved in Aryldialkylphosphatase activity, adenosylhomocisteinase activity, trialkylsulfonium hydrolase activity, acetate-CoA and CoA ligase activity, isocitrate dehydrogenase activity, and hydrolase activity acting on ether bond. In addition, pathways enriched with the correlated gene with *ACE2* are also presented as a bar plot (Figure 2D). From this plot we could suggest that the genes of the *ACE2*-correlated network have a main role in acetate conversion to Acetyl-CoA, leucine degradation and cholesterol biosynthesis.

**Figure 3:**
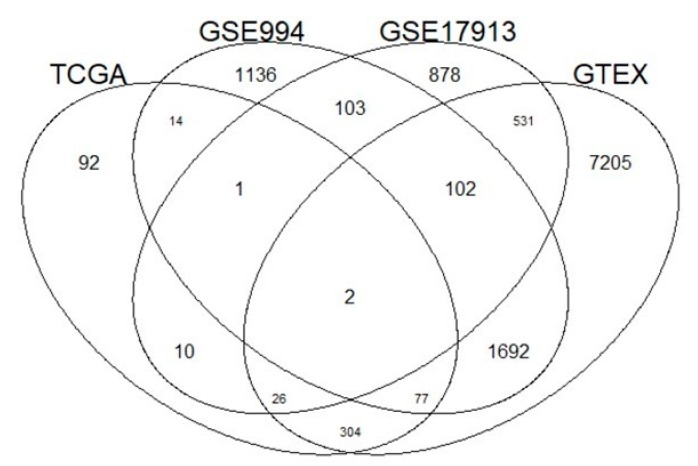
Venn diagram of genes correlated with *ACE2* expression levels in four different datasets (The Cancer Genome Atlas (TCGA), GSE994, GSE17913 and Genotype-Tissue Expression (GTEx)). Ninety-four genes are reported by TCGA-LUAD and GSE994, 39 genes between TCGA-LUAD and GSE17913 and 409 common genes between TCGA-LUAD and GTEx.

Eighty-three percent of the genes (435/526) that correlated with *ACE2* in TCGA were also correlated in at least one of the other three independent datasets (GSE994, GSE17913 and GTEx, *p*-value < 0.05) (Figure 3). In particular, we found 94 common genes between TCGA-LUAD and GSE994, 39 common genes between TCGA-LUAD and GSE17913 and 409 common genes between TCGA-LUAD and GTEx.

Among the top 10 most significant genes correlated to *ACE2* in TCGA-LUAD dataset (Table 1), two genes (*CRIP2* and *ACACA*) were also found in GSE994, ACSL5 was found in GSE17913 and *LRRK2*, *HSD17B4*, *EPHX1*, *MCCC2*, *GSTA4*, *HGD*, and *ROS1* were found in GTEx dataset.

We generated a PPI network considering the direct interactions among correlated genes. We obtained a network of 193 genes and 222 interactions.

**Figure 4:**
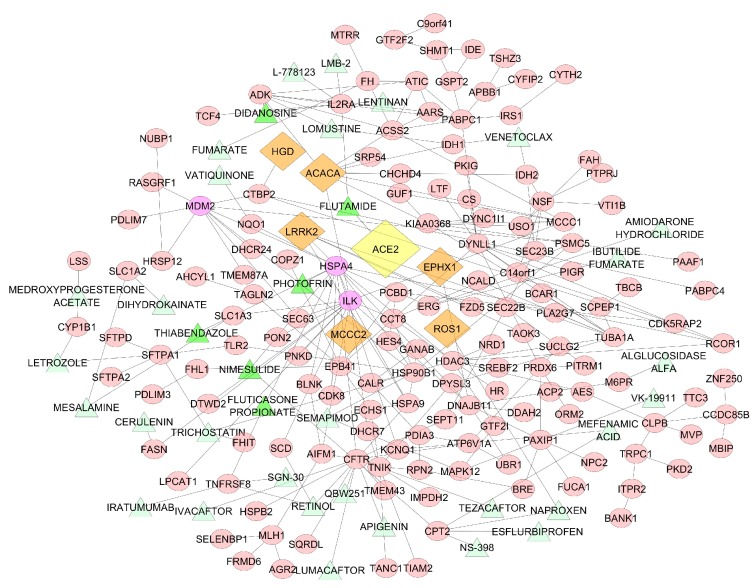
The figure shows the relationships between correlated genes (pink ellipse) with ACE2 (yellow diamond) and known drugs (green triangle) in a protein-protein interaction network. Top genes correlated with ACE2 are represented with orange diamond. Dark green triangles indicate the drugs associated with the genes with a high degree centrality (purple ellipse).

Starting from 7338 drugs originated by the Matador and DGIdb database, we evaluated if drug target genes were overrepresented in the network. We obtained 36 drugs that could influence the densest network (Figure 4).

In this network, *ILK*, *HSPA4*, *DYNLL1* and *MDM2* had a central role with a degree centrality of 17, 13, 11 and 10, respectively. *ILK*, *DYNLL1* and *MDM2* appeared to have no direct significant relationships with known drugs. *HSPA4* was a direct target of Nimesulide, Fluticasone Propionate, Thiabendazole, and Photofrin.

## 4. Discussion

As SARS-CoV-2 is suspected to use ACE-2 protein to enter in the lung cells, we analysed the network of co-expressed proteins with ACE2 in order to define different target genes on which the known drugs could affect the SARS-CoV-2 activity.

Among the top 10 genes with a more significant p-value, we found that nine genes (*LRRK2*, *ACSL5*, *HSD17B4*, *EPHX1*, *MCCC2*, *GSTA4*, *ACACA*, *HGD* and *ROS1*) positively correlated with *ACE2* and *CRIP2* as an *ACE2*-negatively correlated gene.

Leucine rich repeat kinase 2 (LRKK2), involved in Parkinson’s disease, has a role with ACE2 in the reactive oxygen species metabolic process. Moreover, the analysis of the LRRK2 knockout model revealed that this enzyme is involved in ribosomal function, in particular in the regulation of the expression of genes of the clathrin-mediated endocytosis and the integrin for the cell adhesion [31]. Several of the other top ten genes are involved in the fatty acid metabolism, such as Acyl-CoA synthetase long-chain family member 5 (ACSL5) or Hydroxysteroid 17-Beta Dehydrogenase 4 (HSD17B4). Fatty acids are important in the regulation of the fluidity of the movement of receptors of the plasma membrane on the cell surface. Other genes among the top ten are involved in the catabolism of amino acids, such as the Methylcrotonoyl-CoA Carboxylase 2 (MCCC2) enzyme involved in the catabolism of proteins containing Leucine, homogentisate 1,2 dioxygenase (HGD) or Acetyl-CoA Carboxylase Alpha (ACACA). The amino acid catabolism is important for the Acetyl-CoA synthesis during fatty acid metabolism. We also found microsomal Epoxide hydrolase 1 (EPHX1) to have a role during xenobiotic detoxification of exogenous chemicals (tobacco smoke) [32], and Glutathione S transferase (GSTA4), an enzyme of the glutathione metabolism, already described to be altered in influenza virus and respiratory syncytial virus infections [33].

### 4.1. Gene Ontology and Pathway Enrichment Analysis 

Gene Ontology and pathway enrichment analysis for genes correlated to *ACE2* was performed (Figure 2). The results of GO analysis indicated that the 526 genes co-expressed with *ACE2* were mainly enriched in biological processes that belong to the sterol biosynthetic process, multicellular organism protein metabolic process, D-aspartate transport and import and response to unfolded protein (Figure 2A).

The sterol biosynthetic process is essential for the entry of several RNA viruses into the lung cells [34]. It has been already proved that all the agents able to inhibit cholesterol synthesis or block its motility are able to reduce infection by providing membrane rigidity or reducing the permeability [35].

Regarding aspartate transport, aspartate is a charged amino acid that needs specific transporters to be expressed on the cell surface. In tumors, it has been suggested that aspartate availability, related to the presence of the aspartate/glutamate transporter on cell surface, is negatively correlated with the expression of hypoxia molecules [36]. It is also known that asparagine is important for the synthesis of aspartate within the cells and is an important source of nitrogen for pathogens [37].

The unfolded protein response (UPR) is an adaptive condition aimed to rebalance endoplasmic reticulum homeostasis after cellular stress. This process is activated by the host cell as a response to the viral infection to limit the infection. The virus, for its part, tries to manipulate the UPR to support its infection [38]. The final effect of this double control is that the URP is often manipulated during viral infections to aid in the elimination or invasion of the virus.

The results of GO for the cellular component indicated that the genes correlated with *ACE2* were significantly enriched in vacuolar parts and membranes (Figure 2B). In the varicella-zooster virus a role for cytoplasmic vacuoles has been observed [39]. It has been suggested that the transport of viral particles from the membrane of the host cells within the cells could occur by passing through vacuoles by a non-selective bulk-flow transport mechanism [39].

Finally, GO molecular function ontology analysis (Figure 2C) revealed that, among others, there is Aryldialkylphosphatase activity, adenosylhomocysteinase activity, trialkylsulfonium hydrolase activity, acetate-CoA and CoA ligase activity, isocitrate dehydrogenase activity, and hydrolase activity acting on ether bond.

The Aryldialkylphosphatase activity is an enzymatic activity possessed by the paraoxonase (PON) gene family. This enzymatic activity is important for the protection of the cells from oxidative damage for the lipid oxidation metabolism and for the innate immunity response. It is reduced during hepatitis virus B infection and correlates with the functional status of the liver [40]. The inhibition of adenosylhomocysteinase activity, obtained by adenosine dialdehyde, has been described as a potent inhibitor of vaccinia virus early protein synthesis and viral mRNA methylation, suggesting that adenosylhomocysteinase activity is important in the translation of viral proteins and virus replication [41]. Trialkylsulfonium hydrolase activity is one of the enzymatic activities required for the viral replication [42]. Increase in lipid synthesis is essential for viral infection. It has been demonstrated that lipid reduction impacts viral infection (e.g., human cytomegalovirus) [43]. In this process, the central precursor for lipid biosynthesis is cytosolic acetyl CoA (Ac-CoA) [43]; this is the reason why the pathway analysis (Figure 2D) indicated acetate conversion (into acetyl-CoA) as an important ACE2-related pathway. In the same way, leucine degradation could become a source of Acetyl-CoA and acetoacetate by β-hydroxy-β-methylglutaryl-coenzyme A (HMG-CoA) activity, intermediate also of cholesterol biosynthesis [44], described in the pathway analysis. In addition, the results of pathway analysis showed that the genes were enriched in the cholesterol biosynthesis. The role of cholesterol is essential as the entry of pathogenic viruses into the cell is favoured by the presence of cholesterol on the eukaryotic host cells, as discussed before [45].

### 4.2. High Degree Centrality Gene Study and Potential Drug Therapy

We constructed a protein-protein interaction network of the genes correlated with *ACE2*. We obtained a network of 193 genes and 222 interactions. The genes with the highest degree centrality and therefore most involved in the network were *HSPA4*, *ILK* and *MDM2*.

During viral infection, several genes are modulated to allow the attachment and entry of the virus by endocytosis or import/export and translation, which is important for the production of new viral particles. Several studies indicated that heat shock proteins (HSP) are upregulated by viral infection, but also annexin 1 and 2, AKT serine/threonine kinase (AKT1) and hypoxia-inducible factor (HIF1a). In the case of the influenza A virus, HSPs are important for the induction of polymerase activity to induce nuclear import and export and assembly of viral proteins during viral replication [46,47]. Moreover, the heat shock protein A4 (HSPA4) expression is upregulated in patients affected by hepatitis B virus (HBV)-related hepatocellular carcinoma [48], in particular in those with earlier recurrence. The chaperone dynelin LL1 (DYNLL1) has an essential role in the cytoplasmic translocation of hepatitis B viral caspide [49]. All of these reasons make these proteins important targets for the development of new antiviral agents.

Integrin linked kinase (ILK) encodes a protein with a kinase-like domain and four ankyrin-like repeats. It encodes for a protein involved in the cytoplasmic domain of beta integrins. It has been demonstrated for viral myocarditis, a disease that causes sudden cardiac death in children and young adults, ILK inhibition improves the viability of infected cells while blocking viral replication and virus release [50].

MDM2 inhibitors have been already proposed for the development of lymphoma driven by Epstein-Barr virus EBV nuclear antigen-1 (EBNA1) [51]. The inhibition of MDM2 could be also effective in a neuroendocrine carcinoma of the skin called Merkel cell carcinomas, which is caused by Merkel cell polyomavirus. The double inhibition of MDM2 and MDM4 could activate p53, leading to apoptosis of virus-infected cells [52].

Although several recent papers have come out on the possible drug treatment options for COVID-19 and several clinical trials are proposing different compounds for the treatment, we haven’t found any drugs among those that we’d propose, either in clinical trials or in other publications. Recently, an in silico approach enlarged to all possible gene networks altered by COVID-19 disease found several potential therapeutic molecules [53], and although they found some compounds with anti-inflammatory properties, none of them were common with the ones that we found. This is possibly due to the fact that the authors did not consider the ACE2-associated network alone, but combined multiple drugs affecting several members of the gene network of SARS-CoV-2. Nevertheless, in the supplementary material of that paper the authors found among the possible efficient drugs Nimesulide, Fluticasone Propionate, Thiabendazole, and Didanosine, but not photofrin.

Nimesulide belongs to the class of sulphonamides; it is a non-steroidal anti-inflammatory drug (NSAID), which preferentially inhibits the enzyme cyclooxygenase-2 (COX-2). This enzyme takes part in the synthesis of prostaglandin, produced in the course of the cascade of the inflammation process and has relation to the pathogenesis of pain, inflammation and fever. Fluticasone propionate is a new generation corticosteroid used in asthma treatment [54]. Its activity is due to the diffusion of the molecule into the pulmonary cells where it binds to cytoplasmic receptors. The binding to the receptor activates the translocation of the complex into the nucleus where it inhibits cytokine-induced production of proinflammatory proteins. This leads to the suppression of inflammatory mediators, reducing the number of mast cells and lymphocytes [55]. Thiabendazole is an effective antifungal agent and it has been proposed that the drug may have anti-inflammatory properties also in the case of viral infection [56]. Porfimer sodium (photofrin) is a photosensitizing agent used in photodynamic and radiation therapy of tumors; this therapeutic approach has also been proposed in the case of viral infection treatment [57].

In our network, didanosine has adenosine kinase (ADK) and interleukin-2 receptor antagonist (IL2RA) as targets. Didanosine is a dideoxynucleoside analogue used in HIV treatment. The molecule is internalized and is phosphorylated into the active antiviral compound. In this form, it inhibits HIV reverse transcriptase and terminates pro-viral DNA chain elongation [58]. ADK is the key regulatory enzyme of adenosine synthesis. ADK phosphorylates cytokinin nucleosides, maintaining a pool of bioactive cytokinins through interconversion of nucleosides and nucleotides. Cytokinin availability increases the susceptibility of the cells to viral infection, as demonstrated in [59]. The antiviral activity of didanosine could be mediated by interference with ADK activity.

The same target of Didanosine, IL2RA, with *ACE2* is also a direct target of Flutamide. This drug is a specific androgen receptor (AR) antagonist. Its prolonged treatment caused alteration in the renin-angiotensin system without affecting *ACE2* expression levels [60]. This drug is able to block AT2R, evoking vasorelaxation, natriuresis, antigrowth, and anti-inflammatory effects [61]. The activity of flutamide on the ACE2-related network could be mediated by the regulatory role of AR on *ACE2*. Indeed, epidemiological studies found that different sex and age groups in the Chinese population have different susceptibility to SARS-CoV-2 infection due to higher expression of *ACE2* in Asian females compared to males. The induction by estrogen and androgen of *ACE2*, and possibly of *IL2RA*, established a negative correlation between *ACE2* expression, age of the subjects and COVID-19 fatality at both population and molecular levels [62].

Cytokine-based therapies have been developed for cancer treatments, in particular human T cell growth factor, IL-2, has a main role in regulation of growth, differentiation and activation of tumor infiltrating lymphocytes (TLs) such as T cells and natural killers. IL-2RA are agents inhibiting the proliferation of T cells and therefore cytokines release, leading to reduction of inflammation. In Ebola infection, Ebolavirus causes severe dysregulation of the innate immune response resulting in a cytokine storm, and this effect correlates with severe disease and fatal outcomes. Several studies found that hypersecretion of interleukin 1 receptor antagonist (IL-1Ra) was associated with a fatal outcome of Ebola cases: in the COVID-19 infection, it is possible that the hypersecretion of IL-2RA could be blocked by didanosine, leading a decrease of immune response.

## 5. Conclusions

In this study, using public datasets of gene expression profiles we identified new functions and mechanisms of *ACE2*-correlated genes, the putative mediator of SARS-CoV-2 entrance in the cells.

We found a protein-protein interaction network of 193 genes, 22 interactions and 36 potential drugs for future treatment strategies including Nimesulide, Fluticasone Propionate, Thiabendazole, Photofrin and Didanosine. Unexpectedly, among the potentially active drugs only Didanosine is a real antiviral drug, while the others are mostly anti-inflammatory.

However, further studies are needed to validate in the laboratory the role of these drugs for COVID-19 treatment.

## Figures and Tables

**Table 1:** Top 10 genes correlated to *ACE2.*

Name	Function	Gene Ontology	Drug	*p*-value	Correlation
Leucine-Rich Repeat Kinase 2 (LRRK2)	It is involved in multiple activities such as neuronal plasticity, autophagy, and vesicle trafficking	MAPK cascade	Tamoxifen	2 × 10^−8^	0.66
Acyl-CoA Synth. Long Chain Family Memb 5 (ACSL5)	It participates in lipid biosynthesis and fatty acid degradation	long-chain fatty acid metabolic process		3 × 10^−8^	0.65
Cysteine Rich Protein 2 (CRIP2)	It is involved in the differentiation of smooth muscle tissue	protein binding		5 × 10^−8^	−0.64
Hydroxysteroid 17-Beta Dehydrogen. 4 (HSD17B4)	It plays a role in the peroxisomal beta-oxidation pathway for fatty acids	very long-chain fatty acid metabolic process		1 × 10^−7^	0.63
Epoxide Hydrolase 1 (EPHX1)	It participates in the metabolism of lipids	epoxide hydrolase activity	Carbamazepine, Clofibrate, Phenobarbital, AR9281	4 × 10^−7^	0.60
Methylcrotonoyl-CoA Carboxylase 2 (MCCC2)	It is involved in the leucine and isovaleric acid catabolism	protein binding		7 × 10^−7^	0.60
Glutathione S-Transferase Alpha 4 (GSTA4)	It plays a role in cellular defense against oxidative stress	glutathione transferase activity		9 × 10^−7^	0.59
Acetyl-CoA Carboxylase Alpha (ACACA)	It participates in fatty acid synthesis	tissue homeostasis	metformin	4 × 10^−6^	0.56
Homogentisate 1,2-Dioxygenase (HGD)	It plays a role in the catabolism of the amino acids	protein binding		5 × 10^−6^	0.56
ROS Proto-Oncogene 1, Rec. Tyros. Kinase (ROS1)	It contributes in epithelial cell differentiation	regulation of cell growth	Crizotinib Brigatinib Entrectinib Cabozantinib Ceritinib Lorlatinib Foretinib Naproxen Asp-3026 Tae-684 Imatinib	6 × 10^−6^	0.55

## Supplementary Materials

The following are available online at https://www.mdpi.com/1999-4915/12/4/404/s1.
